# Rheumatoid synovial fibroblasts differentiate into distinct subsets in the presence of cytokines and cartilage

**DOI:** 10.1186/s13075-016-1156-1

**Published:** 2016-11-18

**Authors:** Adam P. Croft, Amy J. Naylor, Jennifer L. Marshall, Debbie L. Hardie, Birgit Zimmermann, Jason Turner, Guillaume Desanti, Holly Adams, Adrian I. Yemm, Ulf Müller-Ladner, Jean-Michel Dayer, Elena Neumann, Andrew Filer, Christopher D. Buckley

**Affiliations:** 1 Rheumatology Research Group, Institute of Inflammation and Ageing, University of Birmingham Research Laboratories, Queen Elizabeth Hospital Birmingham, Edgbaston, Birmingham, B15 2WB UK; 2 Department of Internal Medicine and Rheumatology, Justus-Liebig-University Giessen, Kerckhoff-Klinik, Bad Nauheim, Germany; 3 Faculty of Medicine, Centre Médical Universitaire, Geneva, Switzerland; 4 University Hospitals Birmingham NHS Foundation Trust, Birmingham, UK; 5 Sandwell and West Birmingham Hospitals NHS Trust, Birmingham, UK

## Abstract

**Background:**

We investigated two distinct synovial fibroblast populations that were located preferentially in the lining or sub-lining layers and defined by their expression of either podoplanin (PDPN) or CD248, and explored their ability to undergo self-assembly and transmigration in vivo.

**Methods:**

Synovial fibroblasts (SF) were cultured in vitro and phenotypic changes following stimulation with interleukin (IL)-1β, tumor necrosis factor (TNF)-α, and transforming growth factor (TGF)-β1 were examined. To examine the phenotype of SF in vivo, a severe combined immunodeficiency (SCID) human-mouse model of cartilage destruction was utilised.

**Results:**

SF in the lining layer in rheumatoid arthritis (RA) expressed high levels of PDPN compared to the normal synovium, whereas CD248 expression was restricted to sub-lining layer cells. TNF-α or IL1 stimulation in vitro resulted in an increased expression of PDPN. In contrast, stimulation with TGF-β1 induced CD248 expression. In the SCID human-mouse model, rheumatoid SF recapitulated the expression of PDPN and CD248. Fibroblasts adjacent to cartilage expressed PDPN, and attached to, invaded, and degraded cartilage. PDPN^+^ CD248^–^ SF preceded the appearance of PDPN^–^ CD248^+^ cells in contralateral implants.

**Conclusions:**

We have identified two distinct SF populations identified by expression of either PDPN or CD248 which are located within different anatomical compartments of the inflamed synovial membrane. These markers discriminate between SF subsets with distinct biological properties. As PDPN-expressing cells are associated with early fibroblast migration and cartilage erosion in vivo, we propose that PDPN-expressing cells may be an attractive therapeutic target in RA.

## Background

Rheumatoid arthritis (RA) is characterised by the formation of a hyperplastic and invasive synovium, comprised of an expanded synovial fibroblast population with infiltration of inflammatory cells [1]. Synovial fibroblasts (SF) are key mediators of joint destruction and disease persistence [2, 3]. Despite their fundamental importance, these cells have yet to be directly targeted therapeutically, in part due to a lack of cell-specific markers and a full understanding of their biology and role in the pathogenesis of RA [4].

Fibroblasts within the synovium are a heterogeneous cell population with distinct anatomical and functional phenotypes [1]. During persistent synovial inflammation, SF undergo epigenetic changes, assume a stable, activated phenotype, and are capable of invading and destroying articular cartilage [1, 5]. Rheumatoid but not normal SF are also capable of vascular transmigration from their site of primary origin to distant cartilage sites where they attach and invade cartilage [6, 7].

Using an unbiased screen with a panel of monoclonal antibodies to fibroblast cell surface proteins, we have previously identified two trans-membrane proteins (CD248 and GP38) that are highly expressed in inflamed tissue in both mice and humans [4, 8–11]. The differential expression of these fibroblast cell surface markers lead us to propose that specific subsets of fibroblasts, expressing these markers, may play an important and discordant role in the persistence of inflammation and cartilage erosion in RA [12, 13].

In this study, we demonstrate that CD248 and podoplanin (PDPN) identify distinct subsets of SF. Both markers and their related functions are induced temporarily by cytokine treatment in vitro. Furthermore, re-implantation of these subsets of SF in vivo results in recapitulation of many of their functional properties including anatomical location and ability to invade cartilage.

## Methods

### Patients, human synovium, and cartilage

The clinical features of the patients who donated samples are summarized in Table 1. Synovial tissue was obtained from patients with established RA and healthy individuals by ultrasound-guided synovial biopsy as previously described [14]. Cartilage tissue was obtained from healthy areas of cartilage in patients undergoing joint replacement surgery for osteoarthritis. All patients with RA fulfilled the 1987 revised American College of Rheumatology Criteria for RA. Tissue was snap frozen in liquid nitrogen and 5-μm sections were cut prior to fixation in 4 °C acetone.

**Table 1:** Demographics of patient samples

	Rheumatoid arthritis (*n* = 20)	Non-inflammatory controls (*n* = 7)	Significance
Age (years)	56 (48–64)	58 (46-69)	ns
Female, *n* (%)	9 (45)	3 (42.9)	ns
ESR (mm/h)	46.5 (21–62.5)	–	
CRP (mg/l)	26 (7.5–44.5)	–	
DAS28ESR	5.52 (4.47–6.67)	–	
Symptom duration (weeks)	9 (4.3–36)	–	
IgM-RF positive, *n* (%)	9 (45)	–	
ACPA positive, *n* (%)	12 (60)	–	
RhF and ACPA both positive, *n* (%)	9 (45)	–	

Values are shown as median (interquartile range), except where stated

*ACPA* anti-citrullinated protein antibody, *CRP* C-reactive protein, *DAS28ESR* Disease Activity Score-28 with erythrocyte sedimentation rate (*ESR*), *IgM-RF* immunoglobulin M-rheumatoid factor, *ns* not significant, *RhF* rheumatoid factor

### Isolation of fibroblast lines and cell culture

All tissue culture reagents were purchased from Sigma (St. Louis, MO, USA) unless stated otherwise. Fibroblasts were isolated from synovial tissue as previously described [15]. In brief, tissue samples were minced under sterile conditions and dissociated for 2 h at 4 °C with vigorous shaking. The resulting cell/tissue mixture was washed in fresh medium and cultured until adherent fibroblast colonies became confluent. Fibroblasts were cultured in RPMI 1640 supplemented with 1 mM sodium pyruvate, 1 % nonessential amino acid solution, 10 mM glutamine, 100 U/ml penicillin, 100 g/ml streptomycin, and 10 % fetal calf serum (FCS) incubated at 37 °C and 5 % CO_2_. All experiments were performed with at least five sets of matched primary fibroblast lines between passages 2 and 6. For cytokine stimulation assays, primary SF were seeded in triplicates in six-well plates (100,000 cells/well) and incubated until confluence. The cells were serum-starved in RPMI media supplemented with 2 % heat- inactivated FCS for 6 h prior to the addition of human recombinant cytokines (all from Peprotech, Rocky Hill, USA). Tumor necrosis factor (TNF)-α (10 ng/ml), interleukin (IL)-1β (1 ng/ml), and transforming growth factor (TGF)-β1 (10 ng/ml) were added in low-serum media. The cells were harvested by treatment with acutase after 24, 48 and 72 hours and expression of phenotypic markers were analysed.

### Antibodies and immunoflorescence microscopy

Immunostaining and confocal microscopy were performed as previously described [16–18]. We used a species-specific antiCD248 antibody that has been extensively characterized [11, 18–21]. B1/35 is an IgG1 mouse antihuman monoclonal that specifically recognizes human CD248 and does not crossreact with mouse tissue [11, 20, 21]. The monoclonal antiPDPN (eBioscience clone NZ 3.1; Hatfield, UK) was used for flow cytometry and was directly conjugated to PE, and purified for immunostaining. Antihuman VCAM-1 (mouse, IgG1), CD68 (mouse, IgG1 kappa), and CD90 (mouse IgG1 kappa) were obtained from eBioscience. Isotype controls were obtained from eBioscience and Dako (Ely, UK). Antimouse IgG1-FITC, antimouse IgG2b Cy5, antimouse IgG2a Alexa-555 (all Southern Biotech, Birmingham, AL, USA), antirabbit-Alexa 633, and antiFITC-Alexa 488 (Life Technologies, Paisley, UK) were used as secondary and tertiary reagents. Tissue sections from severe combined immunodeficiency (SCID) mice were analyzed for invasion as previously described [6, 7, 22]. Species-specific antibodies were used to identify human cells and exclude staining of mouse fibroblasts in the tissue sections. Cellular density and cell counts were performed on tile scans at × 10 magnification to include an assessment of all cells. High-magnification imaging (×40) was used to confirm the identify of cells and to analyze the anatomical distribution of cells. The Image J “cell counter” plugin (available at http://rsbweb.nih.gov/ij/plugins/cell-counter.html) was used for cell count analysis (Rasband WS, ImageJ, US National Institutes of Health, Bethesda, Maryland, USA; http://imagej.nih.gov/ij/, 1997–2014).

Images were acquired from one to eight different regions of each tissue section using a Zeiss LSM 510 confocal scanning microscope and ZEN pro 2011 imaging software (Zeiss, Welwyn Garden City, UK). Settings within one staining experiment remained unchanged. For each image, the number of pixels with intensity from 30 to 255 of every fluorescent channel was quantified with ZEN pro 2011 and divided by a manually defined area (μm^2^) only including tissue zones containing cells. The average number of fluorescent pixels with intensity 30–255 per unit area (pixel/UA) from all images within one synovial tissue section was calculated. In addition, two researchers independently assessed the fluorescence level of every marker using a semiquantitative scoring system of grade 0–4 combining staining intensity and number of positive cells. Semiquantitative scores correlated well with unbiased pixel analysis scores (Spearman’s rho >0.7, *p* < 0.001 for all markers; data not shown).

In order to measure and account for any variation between staining of sections during different staining runs, we stained sections from the same patients on each occasion for CD248 and PDPN. Intraclass correlation coefficients (ICC) for CD248 (ICC 0.69) and PDPN (ICC 0.74) reflected good (ICC >0.7) internal consistency.

For immunofluorescence of fibroblast lines, cells from synovial biopsy tissue were seeded onto chamber slides at passage 3 with 3 × 10^4^ cells per well and left for 48 h prior to fixation in acetone for 20 min at 4 °C. Slides were subsequently stored at –80 °C until use. Slides were blocked with 10 % normal goat serum and stained using antibodies as described above. Images were acquired on the Leica DM6000 using Leica Application Suite Advanced Fluorescence (Leica Camera AG, Germany).

### Flow cytometry

Primary SF cell cultures from patients with RA (*n* = 5) were washed with phosphate-buffered saline (PBS) and treated with accutase (Life Technologies) dissociation buffer. Cells were stained with APC-conjugated antihuman CD90 (eBioscience), PE conjugated antihuman PDPN (eBioscience, clone NZ-1.3), and FITC conjugated antihuman CD248 B1/35 (produced in house). Isotype controls (eBioscience) were used to set the gates for positive and negative populations. Fluorescence was measured using the FACSCanto II system (BD Biosciences, Oxford, UK) equipped with DIVA 6.2 software (BD Biosciences), and data were analyzed using FlowJo 8.7.3 software (Tree Star Inc., Ashland, OR, USA).

### Quantitative real-time reverse transcription polymerase chain reaction (RT-PCR)

Cell pellets were assayed by real-time PCR for mRNA expression using TaqMan gene expression assays for human CD248 (Hs.00535586), human PDPN (Hs00366766_m1), and human 18S (Hs.03928985) (Life Technologies). Samples were reverse transcribed using a Cells-to-cDNA kitorkit or TaqMan Reverse Transcription kit (both Life Technologies) with PCR Master Mix and TaqMan Universal Master Mix II reagent (Life Technologies). Assays were run on a 7900HT Real-Time PCR system (Life Technologies) according to the manufacturer’s protocol. Data were obtained as Ct values. Expression values for the gene of interest were normalized to those for 18S ribosomal RNA and were transformed to assume a doubling of product with each PCR cycle, calculated using the comparative threshold cycle (Ct) method and then 2^–delta Ct^ formula, as the Ct gene of interest minus the Ct housekeeping gene. Results were expressed as the fold-change relative to values in the control, untreated samples. Each experiment was performed in duplicate and repeated in five different primary RA synovial fibroblast donor cell lines.

### SCID mouse model of cartilage destruction

Mice used in this study were housed in individually ventilated cages in groups of 3–6 individuals on a 12-h light-dark cycle. Healthy areas from human cartilage were obtained from patients undergoing joint replacement surgery due to osteoarthritis. Rheumatoid arthritis synovial fibroblasts (RASFs) from passage 4–5 were trypsinized and added to pieces of a sterile inert gel sponge (3–4 mm^3^) at a concentration of 1 × 10^5^ cells/sponge. The sponges and pieces of cartilage (1–2 mm^3^) were implanted under the adipose capsule of the kidney or subcutaneously in 4-week-old female SCID mice (Charles River, Harlow, UK). For migration assays a cartilage/sponge-only matrix was implanted on the contralateral side simultaneously. Implants and blood were collected at the indicated time points. Implants were frozen prior to tissue sections (10-μm or 8-μm) being obtained, fixed in acetone, and stained with hematoxylin and eosin. Invasion of RASFs into cartilage was evaluated by a minimum of two observers, who were blinded to the source of the implants, using a semiquantitative five-point scale as described previously [8]. For confocal microscopy and quantification, eight tissue sections were analysed per time point in three independent experiments using three mice.

## Results

### CD248 and PDPN identify distinct fibroblast subsets within the hyperplastic inflamed human synovium

**Fig. 1:**
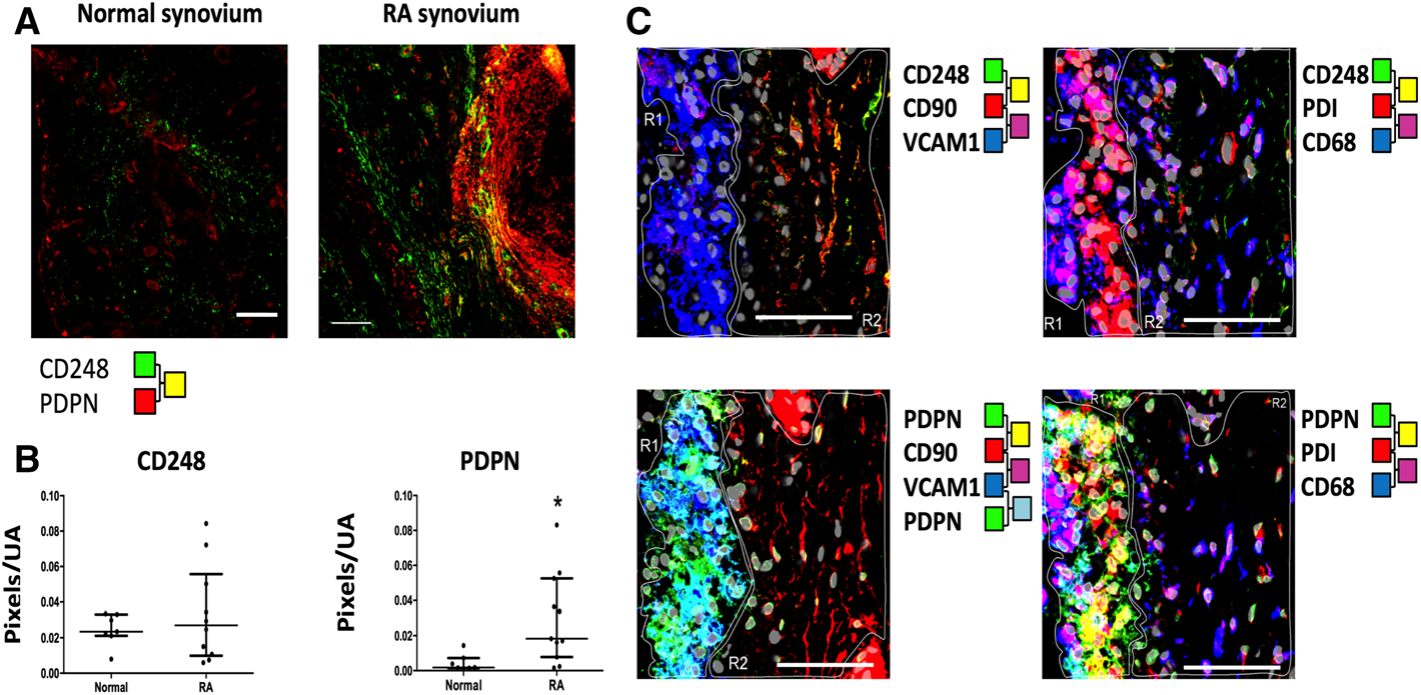
PDPN and CD248 markers identify specific fibroblast subsets localized to distinct anatomical compartments in the inflammatory synovium. **a** Representative confocal images of synovial biopsy tissue from healthy and rheumatoid arthritis (*RA*) synovium. Expression of podoplanin (*PDPN*) and CD248 is shown in healthy (*normal*) compared to rheumatoid synovium. **b** Quantification of marker expression in synovial tissue of patients with RA compared to normal controls. Data are expressed as median and interquartile range for CD248 (*p* = 0.65 by Mann-Whitney *U* test; *n* = 7 normal; *n* = 19 RA) and for PDPN (*p = 0.0013 by Mann-Whitney *U* test; *n* = 7 normal; *n* = 20 RA). **c** High magnification confocal images of rheumatoid synovium showing the lining (*R1*) versus sub-lining (*R2*) layer interface and expression of cell surface markers. *Scale bars* = 100 μm. *PDI* protein disulfide isomerase, *UA* unit area, *VCAM-﻿1﻿* vascular adhesion molecule-1

We first examined the expression of CD248 and PDPN in resting and inflamed synovium using tissue sections of human synovial membrane isolated from healthy individuals and patients with established RA. The expression of PDPN but not CD248 was significantly upregulated in inflamed compared to healthy synovial tissue (Fig. 1a and b). In inflamed tissue, PDPN was mainly expressed by SF in the lining layer whereas CD248 was predominantly expressed by fibroblasts in the sub-lining layer (Fig. 1a). Some PDPN cells could be seen in the sub-lining layer and likely represent lymphatic endothelial cells or perivascular fibroblasts. High-magnification confocal images of rheumatoid synovium in the lining versus sub-lining layer confirmed that CD248 expression was found in association with other known sub-lining layer markers such as CD90, while PDPN expression was found in association with the lining layer markers vascular cell adhesion molecule (VCAM)-1 and protein disulfide isomerase (PDI) (Fig. 1c). Some CD68-positive cells in the lining layer were also PDPN-positive and these cells of monocyte-macrophage lineage have been described previously in mice [23].

### Synovial fibroblasts show phenotypic plasticity

**Fig. 2:**
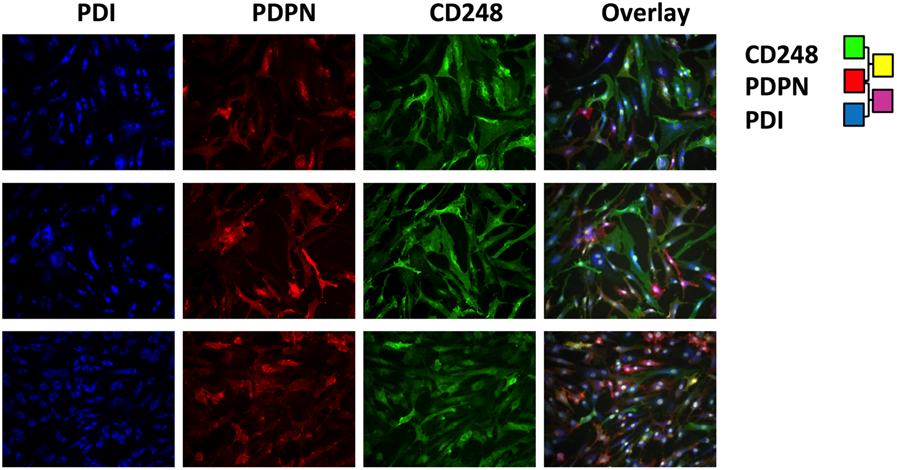
Expression of CD248 and podoplanin (*PDPN*) in cultured RASF. Three representative confocal images of different RASF lines in culture are shown, ×40 magnification: *green*, CD248; *blue*, PDI; *red* PDPN; and *grey*, nuclei. *PDI* protein disulfide isomerase

Using SF from rheumatoid synovial biopsy tissue explants, we next evaluated the expression of PDPN and CD248 in cultured SF using immunofluorescence microscopy. SF in culture were heterogeneous with respect to the expression of PDPN and CD248 (Fig. 2). There were distinct populations of PDPN- and CD248-expressing fibroblasts and only very rarely were cells seen that stained positive for both markers.

To evaluate whether the phenotypic markers observed in vivo were inducible in vitro, SF were cultured in low serum conditions (2 % FCS) with TGF-β1, TNF-α, or IL-1β . These cytokines were chosen as TNF-α and IL-1β have been shown to stimulate fibroblasts to produce large amounts of proteolytic enzymes that are destructive to cartilage and bone [2]. TGF-β1 is also known to be capable of inhibiting the synthesis of metalloproteinases and thereby to inhibit joint damage [2].

**Fig. 3:**
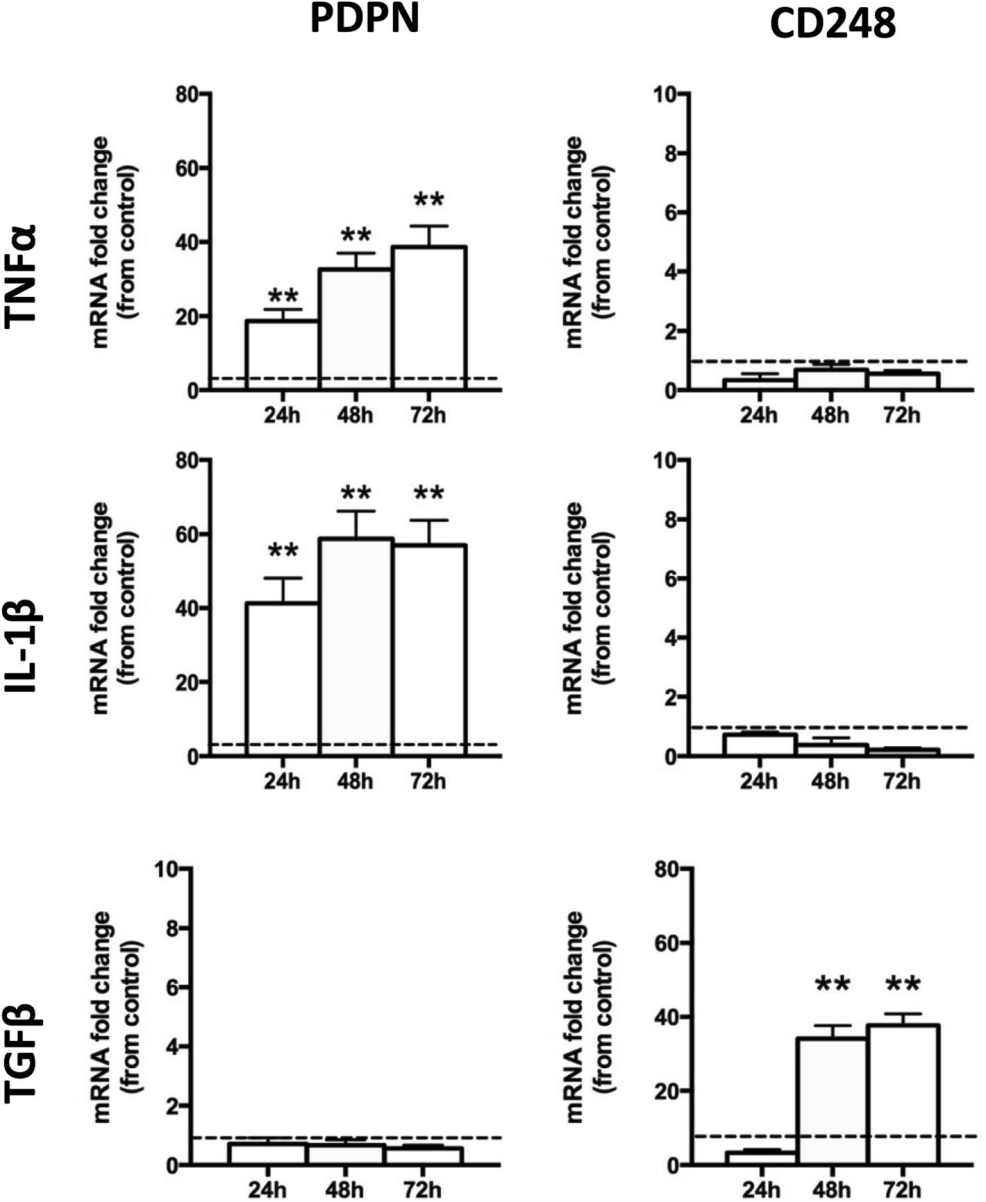
Cytokines stimulate differential expression of CD248 and podoplanin (*PDPN*) in vitro. Expression of PDPN and CD248 by quantitative RT-PCR expressed as mRNA fold-change from unstimulated cells (mean *±* SEM). **P < 0.01 by one-way ANOVA with Bonferroni post hoc analysis. *IL* interleukin, *TGF* transforming growth factor, *TNF* tumor necrosis factor

**Fig. 4:**
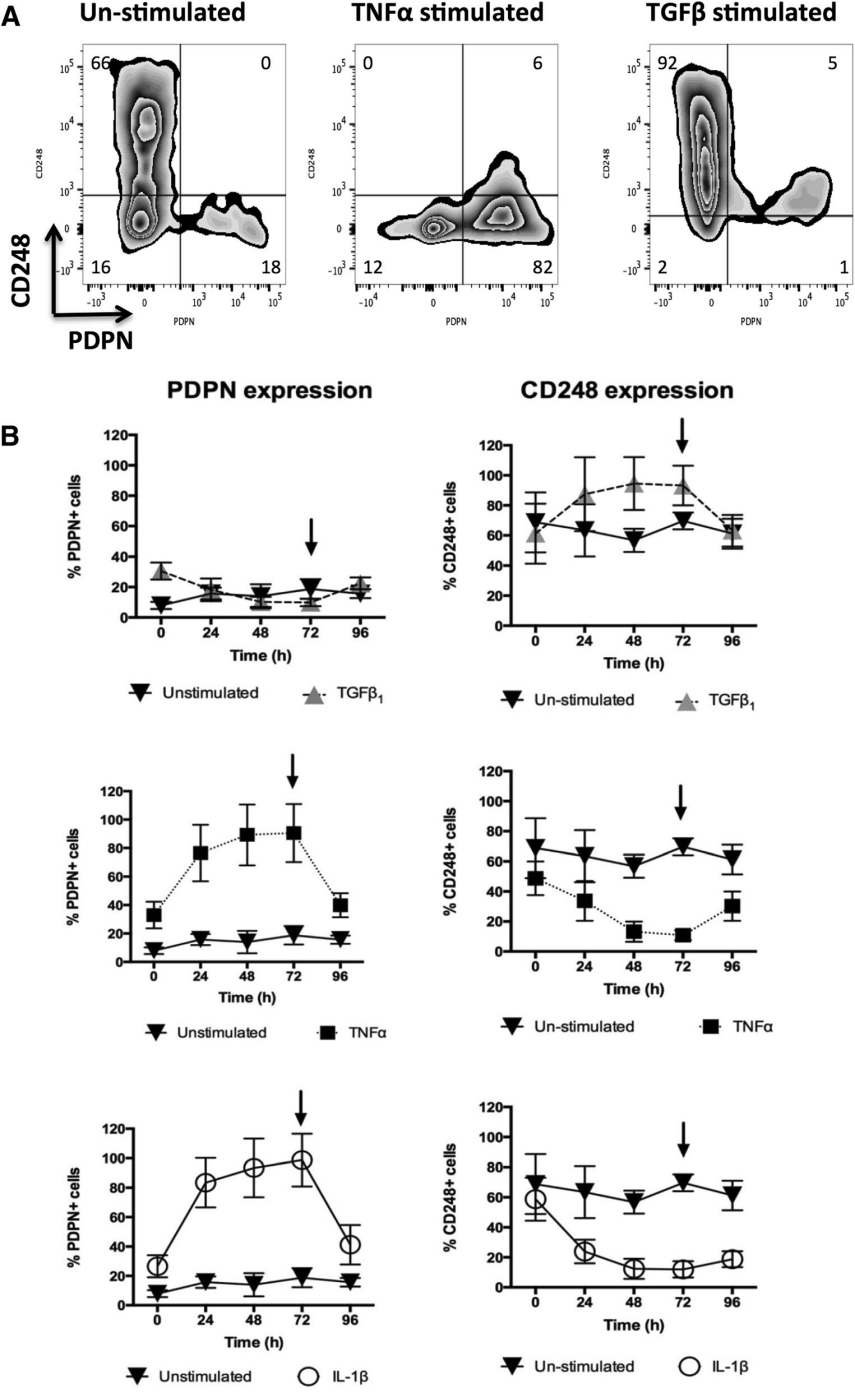
Cytokines stimulate differentiation of RASF towards specific fibroblast subsets in vitro. **a** Dual color flow cytometry of fibroblasts stimulated with either TNF-α or TGF-β in vitro. Histograms of CD248 versus podoplanin (*PDPN*) expression following cytokine stimulation. Percentage of positive cells in each gate is displayed. **b** The percentage of PDPN^+^ and CD248^+^ cells measured by flow cytometry in cultured RASF in response to cytokine stimulation (TNF-α, 10 ng/ml; IL-1β, 1 ng/ml; TGF-β1, 10 ng/ml). Data are expressed as mean *±* SEM of the number of PDPN- and CD248-expressing fibroblasts expressed as a percentage of total cells at each time from *N* = 5 donor cell lines. Representative histograms of flow cytometric quantification of marker expression following cytokine stimulation are shown. *Arrow* indicates removal of cytokine. *IL* interleukin, *TGF* transforming growth factor, *TNF* tumor necrosis factor

Expression of PDPN and CD248 in stimulated SF was assessed using RT-PCR for RNA expression (Fig. 3) and flow cytometry (Fig. 4) for protein cell surface expression. RASF stimulated with either TNF-α or IL-1β upregulated the expression of PDPN at both the mRNA and protein level over 72 h of exposure. In contrast, this treatment did not result in an upregulation of CD248 expression but rather significantly reduced CD248 expression. Stimulation with TGF-β1 resulted in increased expression of CD248 but not PDPN expression at both the protein and mRNA levels. Following seeding at equal plating density, there was no significant difference in the proliferation rate of the two cell populations after 72 h. Continuous cytokine stimulation was required to maintain the expression of these cell surface markers since removal of cytokines at 72 h resulted in a return to the baseline expression of both PDPN and CD248 when evaluated at 24 h post-removal (Fig. 4b).

### Rheumatoid SF self-organize into lining and sub-lining layers in the presence of human cartilage

**Fig. 5:**
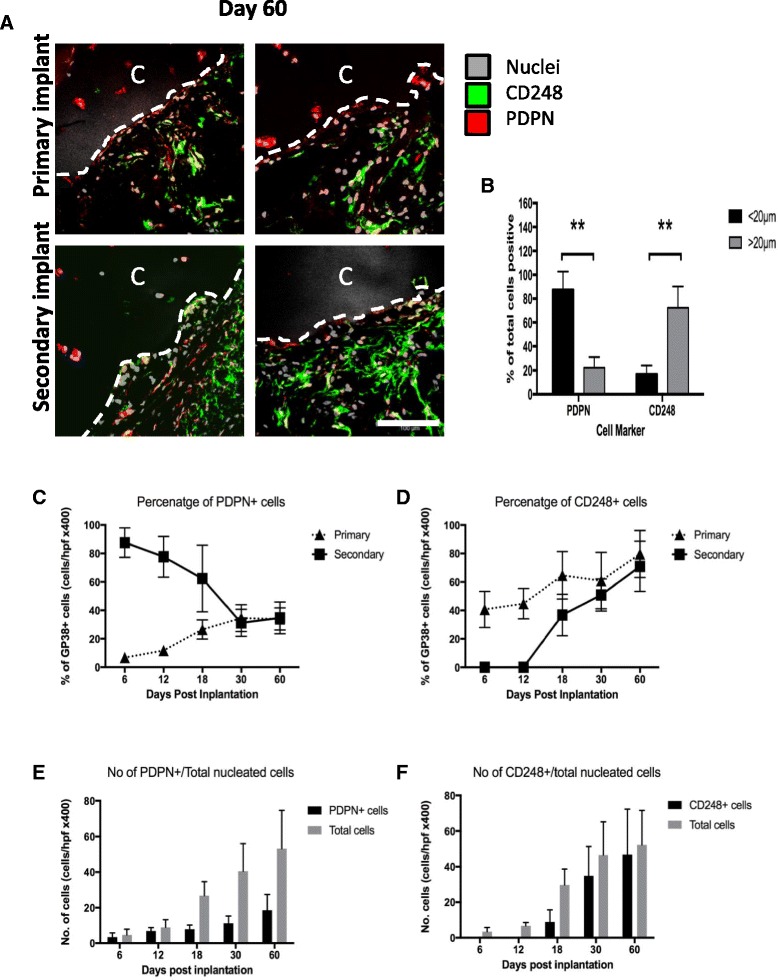
In vivo cartilage destruction and vascular transmigration following implantation of RASF under the kidney capsule of SCID mice. **a** Representative confocal images of tissue sections of the fibro/cartilage matrix harvested 60 days following implantation showing expression of CD248 and podoplanin (*PDPN*). *Dashed line* shows demarcation of the cartilage interface. *Scale bar* = 100 μm. **b** The percentage of cells positive for each marker within 20 μm proximity to cartilage is displayed as the mean ± SEM for each cell marker. Time course analysis of the percentage of total nucleated cells expressing PDPN (**c**) and CD248 (**d**) within the primary implant compared to the secondary (cartilage only) implant at 6, 12, 18, 30, and 60 days following implantation. Absolute numbers of total nucleated cells over time in sections of secondary implant are also shown compared to the total number of those cells positive for each of these markers over time (**e**, **f**). Data are expressed as the mean ± SEM of the percentage of cells expressing each cell marker at each time point from eight tissue sections per implant (*n* = 3 mice). **P < 0.001﻿ . *C* cartilage fragment

We next explored whether PDPN-expressing fibroblasts could invade articular cartilage in vivo using the SCID mouse model of cartilage destruction [5, 6, 24]. RASF were seeded into an inert sponge and co-implanted with healthy human articular cartilage subcutaneously or under the renal capsule of SCID mice. At 60 days post-implantation, in the presence of cartilage alone, RASF self-assembled with a cellular architecture that recapitulated the organization of PDPN and CD248 observed in the inflamed human synovium (Fig. 5a). Fibroblasts proximate to the cartilage (less than 20 μm distance) were PDPN^+^ CD248^–^ and cells beneath this layer (greater than 20 μm from the cartilage) expressed CD248 and rarely expressed PDPN (Fig. 5b) CD248^+^ cells formed an apparent sub-lining layer beneath the layer of PDPN-expressing fibroblasts. SF that had invaded cartilage did not express CD248 but rather expressed PDPN. This occurred despite the fact that the fibroblasts originated from a heterogeneous mix of CD248- and PDPN-expressing RASF (Fig. 2).

Recent studies using the SCID mouse model have shown that SF can migrate from the primary site of implantation to distant sites of exposed cartilage through the vascular system [6]. To analyze whether PDPN^+^ or CD248^+^ fibroblasts were involved in this process, a secondary implant containing cartilage and sponge only was implanted either subcutaneously or under the renal capsule on the contralateral side to the primary fibroblast-containing implant. At 60 days post-implantation there was no significant difference in the invasion score of fibroblasts into cartilage between the primary and secondary implant (2.3 ± 0.9 in the primary implant and 1.8 ± 0.7 in the secondary implant; *p* = 0.56 by Student’s *t* test). There was also no significant difference in the organization of RASF into PDPN^+^ and CD248^+^ cells or the percentage of cells expressing each of these markers between the primary and secondary implant (Fig. 5c–f).

However, there were marked differences in the dynamics of PDPN and CD248 expression on fibroblasts within both implants when observed over time (Fig. 5c). In the primary implant, the percentage and absolute number of fibroblasts positive for PDPN increased between day 6 and day 30 before reaching a plateau. The same pattern was observed for CD248 expression except that the percentage of RASF expressing this marker reached a plateau between day 18 and day 30. In the secondary implant, containing cartilage only, the number of infiltrating cells progressively increased over time. This was coincident with the formation of murine vasculature (as indicated by CD31 expression) in the primary implant at day 6 (data not shown). However, within the secondary implant, at early time points from day 6 onwards, almost all RASF present within the implant expressed PDPN and not CD248. The percentage of cells expressing PDPN in the secondary implant decreased over time, reaching a plateau at day 30. In complete contrast, CD248^+^ cells were only detectable in the secondary implant 18 days post-implantation and increased in percentage up to day 60 (Fig. 5c).

## Discussion

In this study we have utilized two markers (PDPN and CD248) to distinguish between distinct populations of SF defined by anatomical location and function. We demonstrate that RASF have an intrinsic capacity to self-organize into specific lining and sub-lining layers defined by their expression of these markers in the presence of cartilage. Whilst the exact role of these two cell-specific markers in inflammatory arthritis is not fully known, PDPN appears to be expressed in fibroblasts with the capacity to invade and destroy cartilage. This observation matches previous reports suggesting that PDPN upregulation by cancer cells and cancer-associated fibroblasts promote metastasis [25].

CD248 and PDPN are expressed in a highly restricted pattern in diseased tissue. PDPN expression predominately maps to the lining layer of the synovium and is seen only on a small number of cells within the sub-lining layer, whereas CD248 is predominantly expressed by the sub-lining layer RASF. This differential expression is less pronounced in normal tissue where expression of CD248 is low and PDPN expression in the restricted to lymphatic endothelial cells and expressed at low levels in the lining layer. The distinction between lining and sub-lining layer RASF is important when considering that, during chronic inflammation, the lining layer becomes hyperplastic and invades cartilage and bone. Synovial fibroblasts, but not cells of the monocyte/macrophage lineage, have been shown to be the predominant source of collagenase and therefore these cells are key effector cells in joint damage [26].

When a heterogeneous population of RASF were implanted with human cartilage into SCID mice, we found that RASF self-organized into lining and sub-lining layers, in the absence of any other immune cells or stimulus, suggesting that this property is an intrinsic property of SF. Synovial fibroblasts have also been shown to establish a lining layer structure at the interface between matrix and fluid phase in three-dimensional culture [27]. In agreement with our findings of self-organization, this process is not dependent on the presence of other cell types.

Although the molecular basis for the cellular remodeling of the inflamed synovium remains to be fully elucidated, cadherin-11 has been shown to play a critical role in integrating fibroblasts into the synovial lining layer and for the development of an effective hyperplastic synovium during inflammation [27]. Genetic deletion of this molecule inhibits the development of a hyperplastic synovial lining layer and abolishes inflammatory arthritis in vivo. These studies serve to demonstrate the importance of the fibroblast-lining layer in the pathophysiology of inflammatory arthritis [27].

RASF are also capable of transmigration to a secondary site of cartilage implantation as previously described [6]. Following engraftment we found that RASF at the secondary site self-organized into layers of SF indistinguishable from the primary site of implantation. When we evaluated the cells involved at the early stages of engraftment at secondary sites, we found that the majority of SF expressed PDPN and not CD248. It remains unknown if the finding of high PDPN expression at early time points and CD248 at later time points is a temporal effect in which PDPN^+^ RASF migrate early and CD248^+^ RASF migrate later or whether this finding results from the in situ differentiation of PDPN-expressing RASF to a CD248-expressing fibroblast. In support of the latter, we were able to demonstrate plasticity, based on cytokine exposure, in the expression of these markers by fibroblasts in vitro. Definitive evidence for the phenotype of migrating fibroblasts can only be determined by isolating migrating cells in the blood stream. Unfortunately, we were unable to identify human fibroblasts in the blood of mice. This is probably due to the low circulating frequency of these cells in the vasculature.

We found that PDPN expression correlates with an aggressive fibroblast phenotype. Consistent with other studies, PDPN expression was upregulated in the lining layer during chronic inflammation and expression was inducible in vitro in response to pro-inflammatory cytokines. We found that the expression of PDPN and CD248 is associated with functional consequences in vivo*,* since re-implantation in a SCID mouse in the presence of cartilage resulted in PDPN-expressing RASF invading articular cartilage implanted at a secondary site.

RASF are known to acquire an activated phenotype and are capable of invading cartilage (in the absence of other stimuli) even following prolonged culture [1, 5–7, 28]. Interestingly, PDPN was only expressed by cells proximal to the cartilage interface or by cells that had invaded cartilage. This is consistent with a lining layer phenotype. It is therefore likely that many factors play a role in determining the expression of these cell surface markers.

CD248 is transmembrane receptor whose ligands are reported to be extracellular matrix molecules (fibronectin and type I/IV collagen) [29, 30] and is widely expressed on mesenchymal cells in the developing embryo [19, 31]. However, expression is downregulated postnatally but upregulated during tissue remodeling. We found CD248-expressing fibroblasts predominantly in the sub-lining tissue of the inflammatory synovium, consistent with previous studies [32]. As expected from their anatomical location, CD248^+^ SF did not invade the implants of cartilage in vivo. It is not yet known if CD248-expressing fibroblasts are required for the formation of a PDPN-expressing fibroblast layer capable of invading cartilage and bone.

## Conclusion

Based on the observations described in this study, we propose that the expression of PDPN and CD248 can be used to identify subsets of RASF defined by their anatomical location in the inflamed joint and their ability to invade and degrade articular cartilage in vivo. The differential expression of these cell surface markers on fibroblasts make these markers ideal candidates for selectively targeting distinct subpopulations of SF. Better understanding of the function of these molecules, and the impact of selective deletion of cells that express them, will be critical for the development of fibroblast-targeted treatments in the future.

## Abbreviations

FCS: Fetal calf serum
ICC: Intraclass correlation coefficient
IL: Interleukin
PDI: Protein disulfide isomerase
PDPN: Podoplanin
RA: Rheumatoid arthritis
RASF: Rheumatoid arthritis synovial fibroblast
RT-PCR: Reverse transcription polymerase chain reaction
SCID: Severe combined immunodeficiency
SF: Synovial fibroblasts
TGF: Transforming growth factor
TNF: Tumor necrosis factor
UA: Unit area
VCAM-1﻿: Vascular cell adhesion molecule-1
